# Supplementary material for: Global climate gradients structure soil Legionella diversity, relative abundance and pathogen distributions
Source: bioRxiv. 2026 Mar 11:2026.03.10.710744. Preprint. [Version 2] doi: 10.64898/2026.03.10.710744 (PMC13060998; doi:10.64898/2026.03.10.710744)
Supplement: Supplement 2 [file NIHPP2026.03.10.710744v2-supplement-2.pdf]

bioRxiv preprint doi: https://doi.org/10.64898/2026.03.10.710744; this version posted March 11, 2026. The copyright holder for this preprint (which was not certified by peer review) is the author/funder, who has granted bioRxiv a license to display the preprint in perpetuity. It is made available under aCC-BY 4.0 International license.

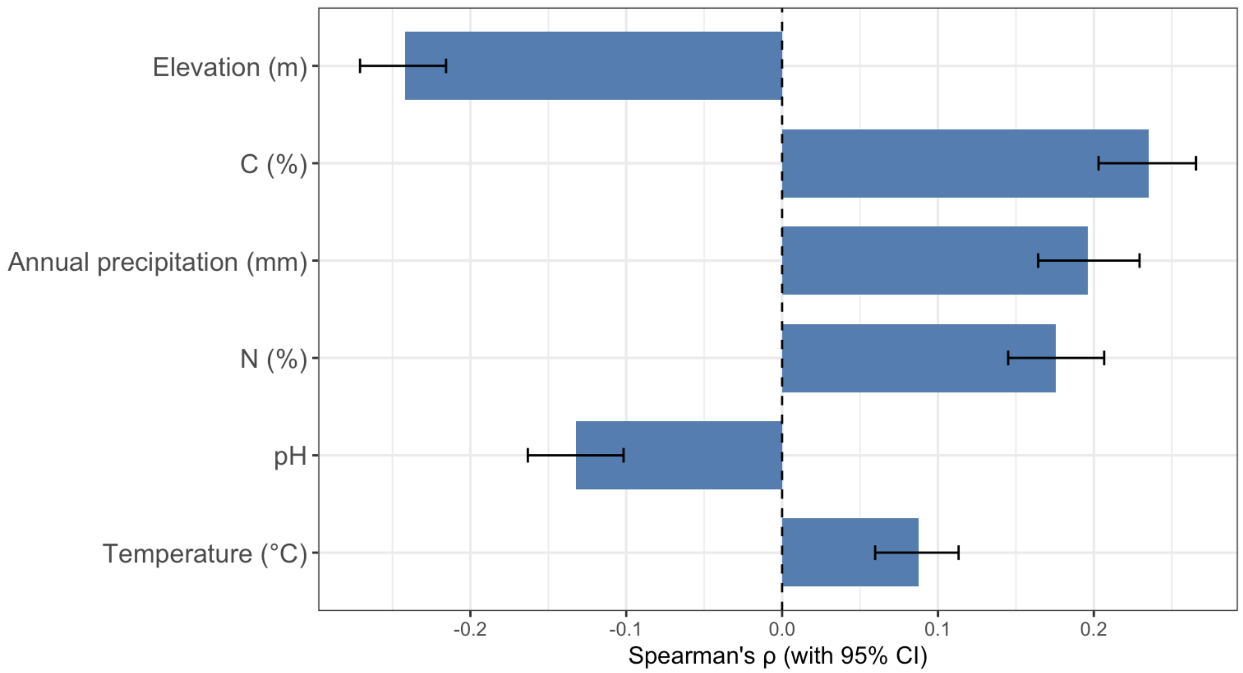

Fig. S1 - Spearman correlations between *Legionella* relative abundance and climatic and biogeochemical variables. Bars represent Spearman's rank correlation coefficients (ρ) between *Legionella* relative abundance and each environmental variable across all samples. Error bars indicate 95% confidence intervals.

bioRxiv preprint doi: https://doi.org/10.64898/2026.03.10.710744; this version posted March 11, 2026. The copyright holder for this preprint (which was not certified by peer review) is the author/funder, who has granted bioRxiv a license to display the preprint in perpetuity. It is made available under aCC-BY 4.0 International license.

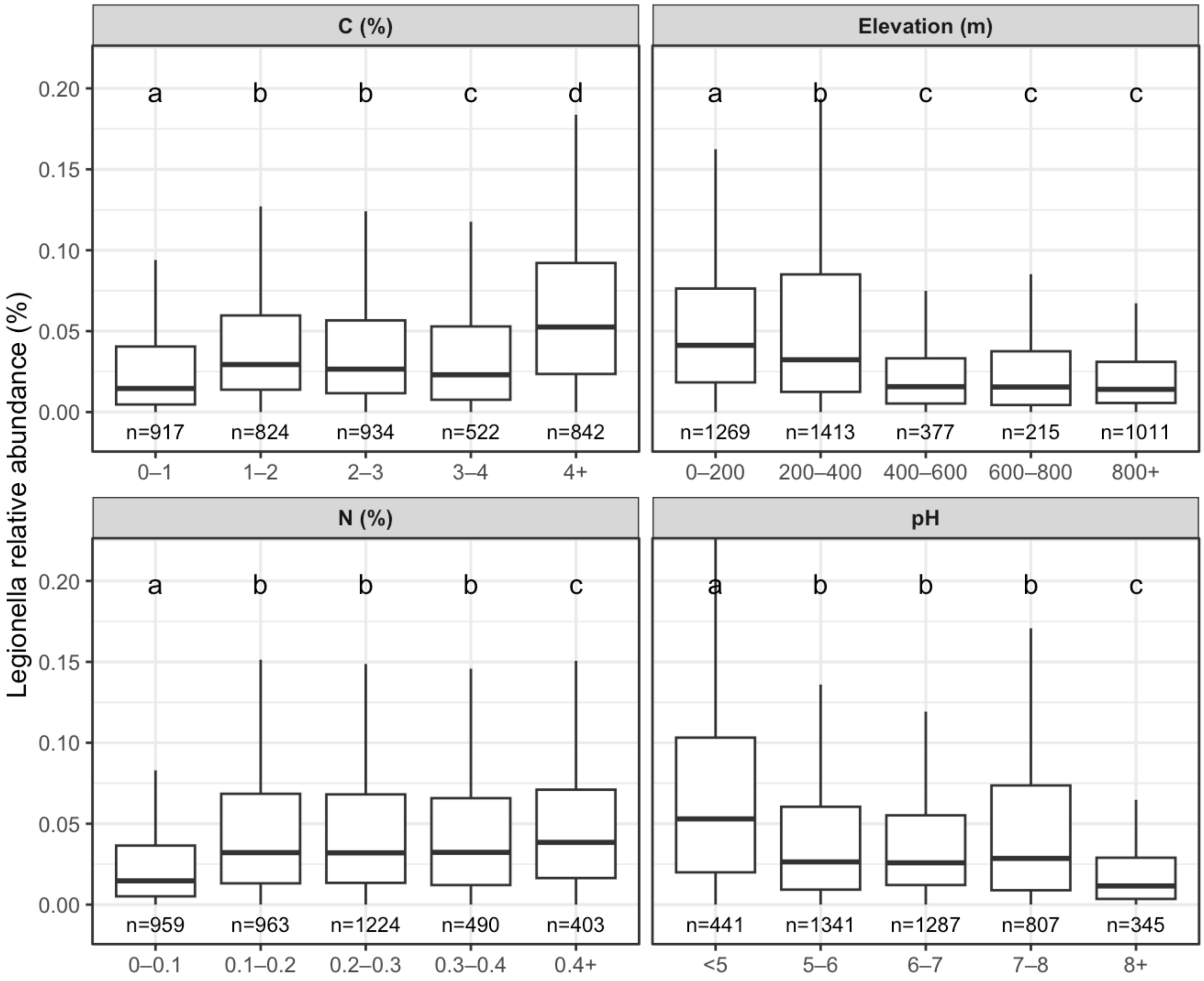

Fig. S2 - Variation in *Legionella* relative abundance across biogeochemical and abiotic gradients. Boxplots show *Legionella* relative abundance across five bins spanning the observed range of soil carbon (C), elevation, nitrogen (N) and pH values. Boxes represent the interquartile range (IQR), center lines indicate medians and whiskers extend to 1.5x the IQR. Sample sizes for each bin are indicated below the x-axis. Letters above boxes denote statistically distinct groups identified using Dunn's post hoc comparisons following Kruskal-Wallis tests ($p<0.05$).

bioRxiv preprint doi: https://doi.org/10.64898/2026.03.10.710744; this version posted March 11, 2026. The copyright holder for this preprint (which was not certified by peer review) is the author/funder, who has granted bioRxiv a license to display the preprint in perpetuity. It is made available under aCC-BY 4.0 International license.

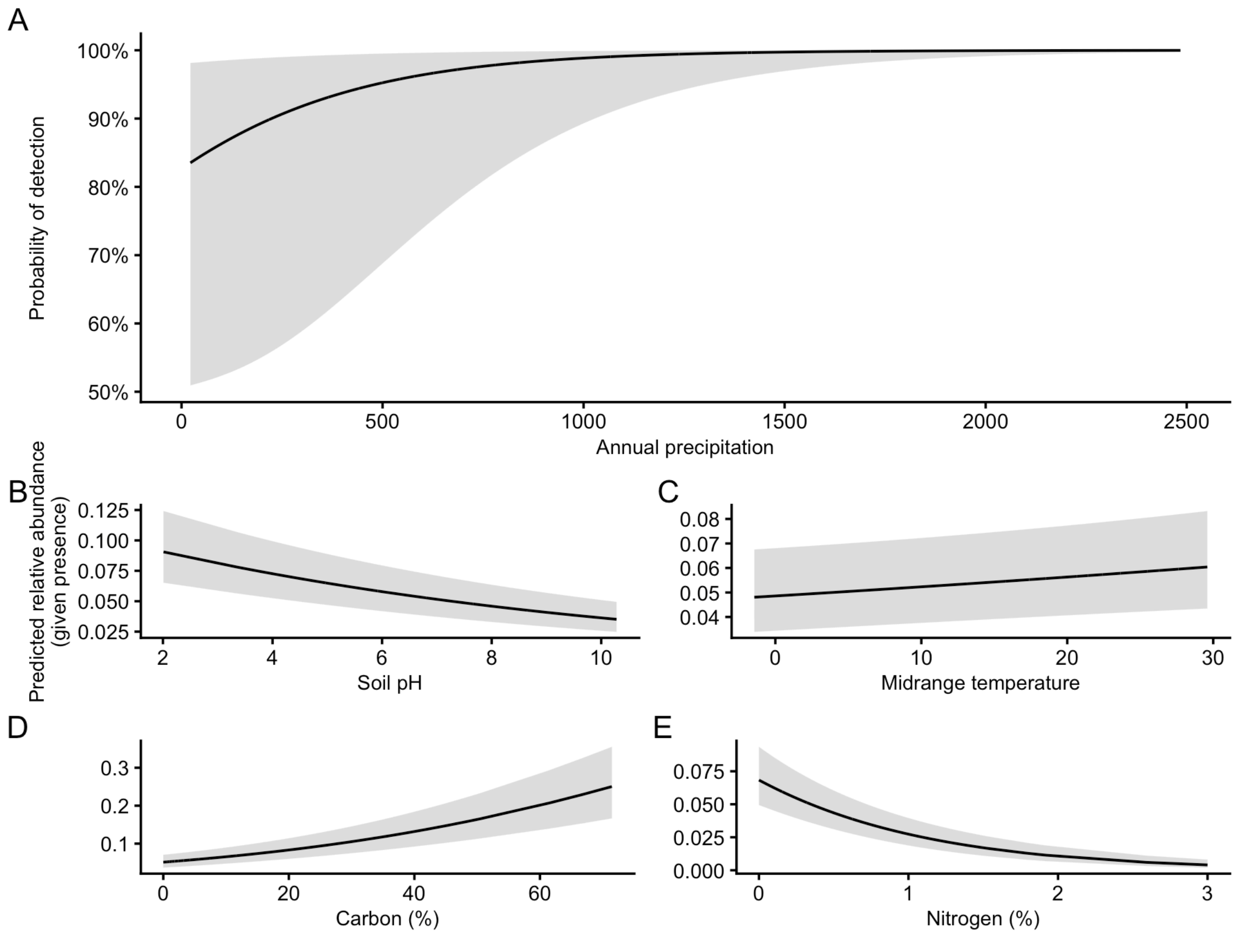

Fig. S3 - Hurdle mixed-effects model results for predictors of *Legionella* occurrence and abundance. (A) Predicted probability of detecting *Legionella* as a function of annual precipitation (mm) from a binomial mixed-effects model. (B-E) Predicted relative abundance of *Legionella* conditional on presence from a beta mixed-effects regression model across gradients of soil pH, midrange temperature, soil carbon (C), and soil nitrogen (N). Lines represent model predictions and shaded regions indicate 95% confidence intervals. Study was included as a random intercept in all models.

bioRxiv preprint doi: https://doi.org/10.64898/2026.03.10.710744; this version posted March 11, 2026. The copyright holder for this preprint (which was not certified by peer review) is the author/funder, who has granted bioRxiv a license to display the preprint in perpetuity. It is made available under aCC-BY 4.0 International license.

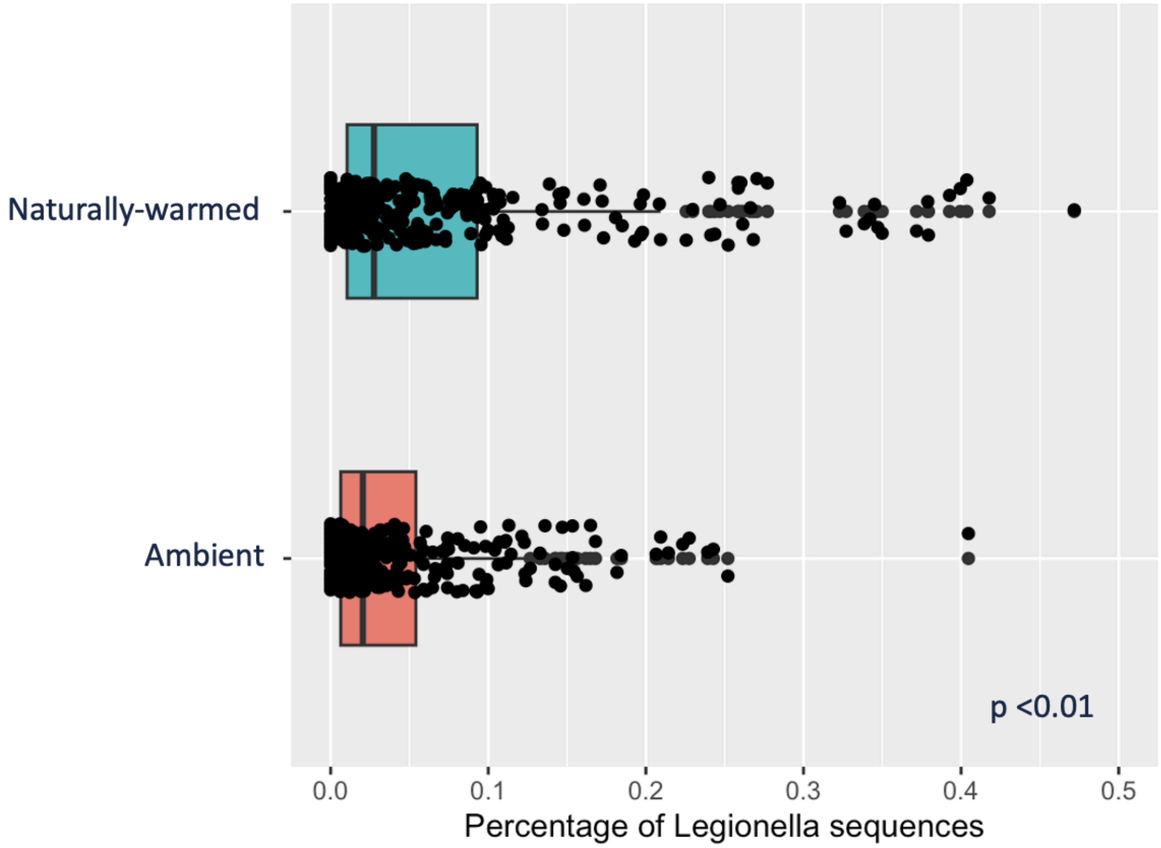

Fig. S4 - *Legionella* relative abundance at geothermally warmed and ambient sites in Iceland. Comparison of *Legionella* relative abundance between naturally warmed soil (+6°C) and nearby ambient sites. Points represent individual samples and boxplots summarize the distribution (median and interquartile range).

bioRxiv preprint doi: https://doi.org/10.64898/2026.03.10.710744; this version posted March 11, 2026. The copyright holder for this preprint (which was not certified by peer review) is the author/funder, who has granted bioRxiv a license to display the preprint in perpetuity. It is made available under aCC-BY 4.0 International license.

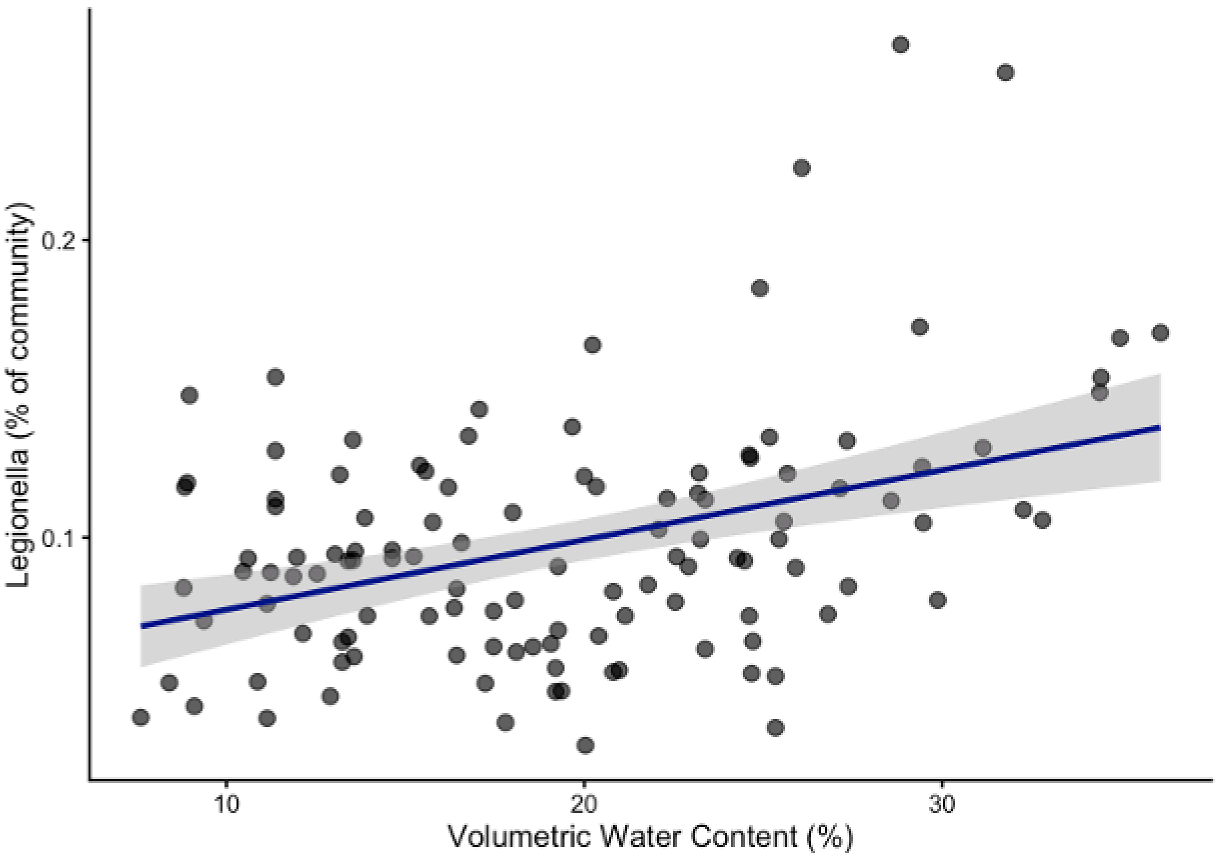

Fig. S5 - Relationship between soil moisture and *Legionella* relative abundance at rainfall-manipulation sites in Wisconsin. Relative abundance of *Legionella* sequences plotted against volumetric water content (%). Points represent individual samples. The line indicates the fitted linear regression and the shaded region shows the 95% confidence interval.

bioRxiv preprint doi: https://doi.org/10.64898/2026.03.10.710744; this version posted March 11, 2026. The copyright holder for this preprint (which was not certified by peer review) is the author/funder, who has granted bioRxiv a license to display the preprint in perpetuity. It is made available under aCC-BY 4.0 International license.

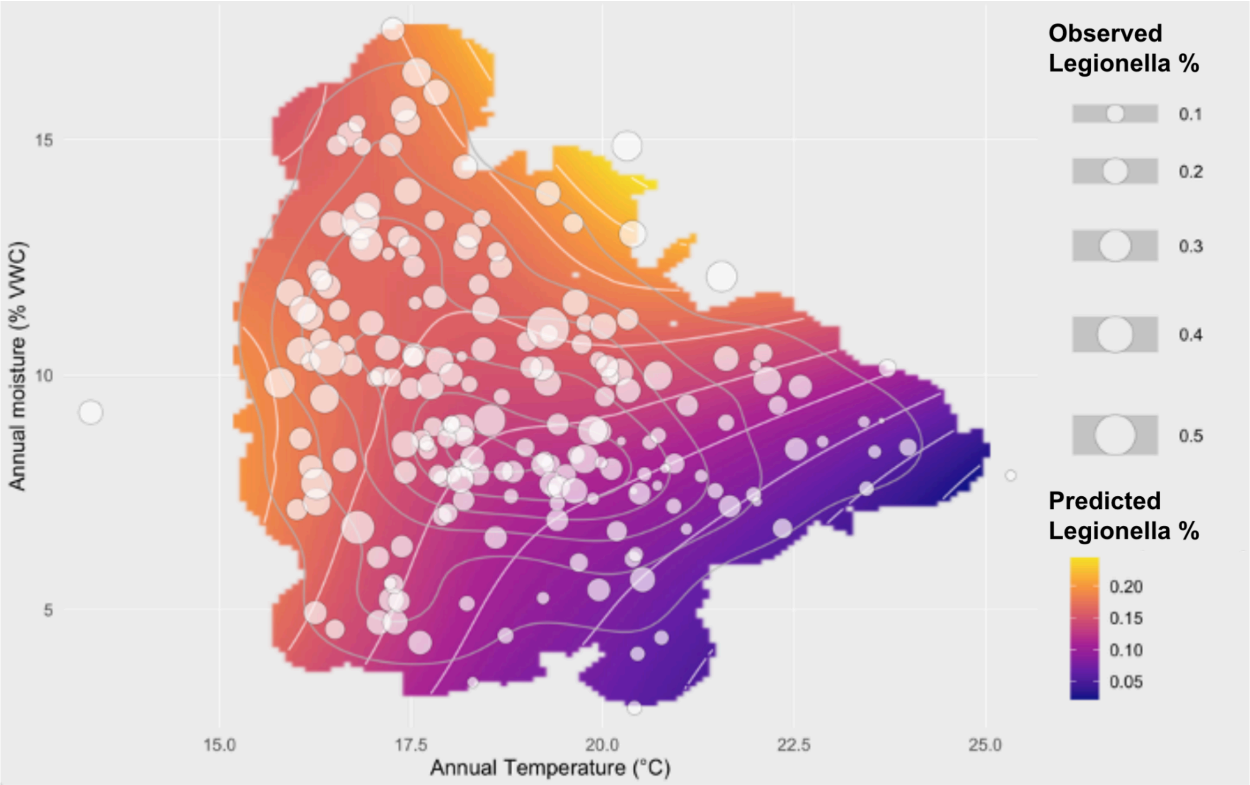

Fig. S6 - *Legionella* relative abundance across temperature and soil moisture gradients at a combined climate-manipulation experiment in Oklahoma. Observed *Legionella* relative abundance is shown as points, with point size proportional to the percentage of *Legionella* sequences detected in each sample. The colored surface represents LOESS-predicted *Legionella* relative abundance, with contour lines indicating predicted abundance levels.

bioRxiv preprint doi: https://doi.org/10.64898/2026.03.10.710744; this version posted March 11, 2026. The copyright holder for this preprint (which was not certified by peer review) is the author/funder, who has granted bioRxiv a license to display the preprint in perpetuity. It is made available under aCC-BY 4.0 International license.

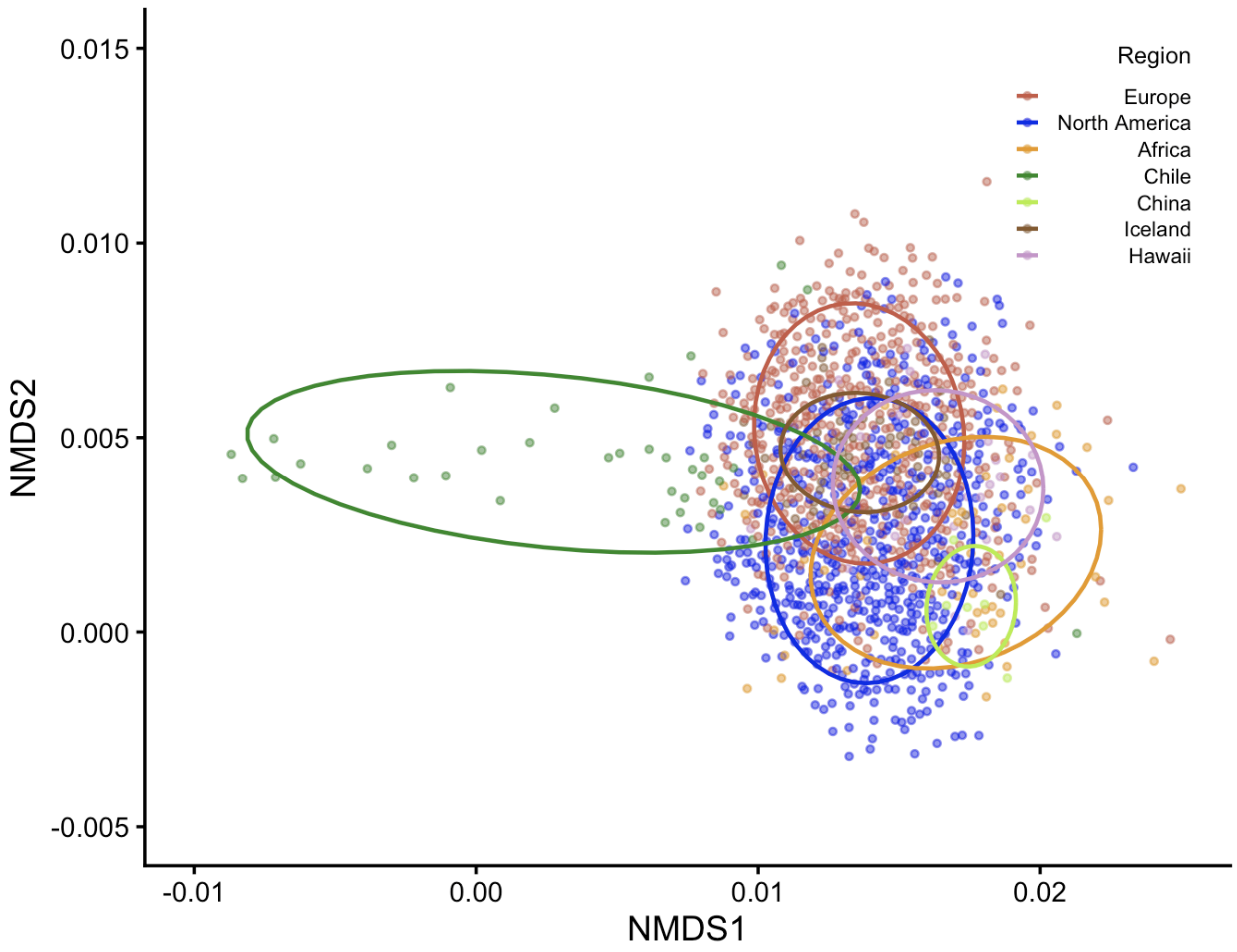

Fig. S7 - Regional structuring of *Legionella* community composition. Non-metric dimensional scaling (NMDS) ordination of *Legionella* community composition based on Bray-Curtis dissimilarities calculated from ASV relative abundances. Each point represents an individual sample and colors indicate geographic region. Ellipses represent 95% confidence intervals around the centroid of each region.

bioRxiv preprint doi: https://doi.org/10.64898/2026.03.10.710744; this version posted March 11, 2026. The copyright holder for this preprint (which was not certified by peer review) is the author/funder, who has granted bioRxiv a license to display the preprint in perpetuity. It is made available under aCC-BY 4.0 International license.

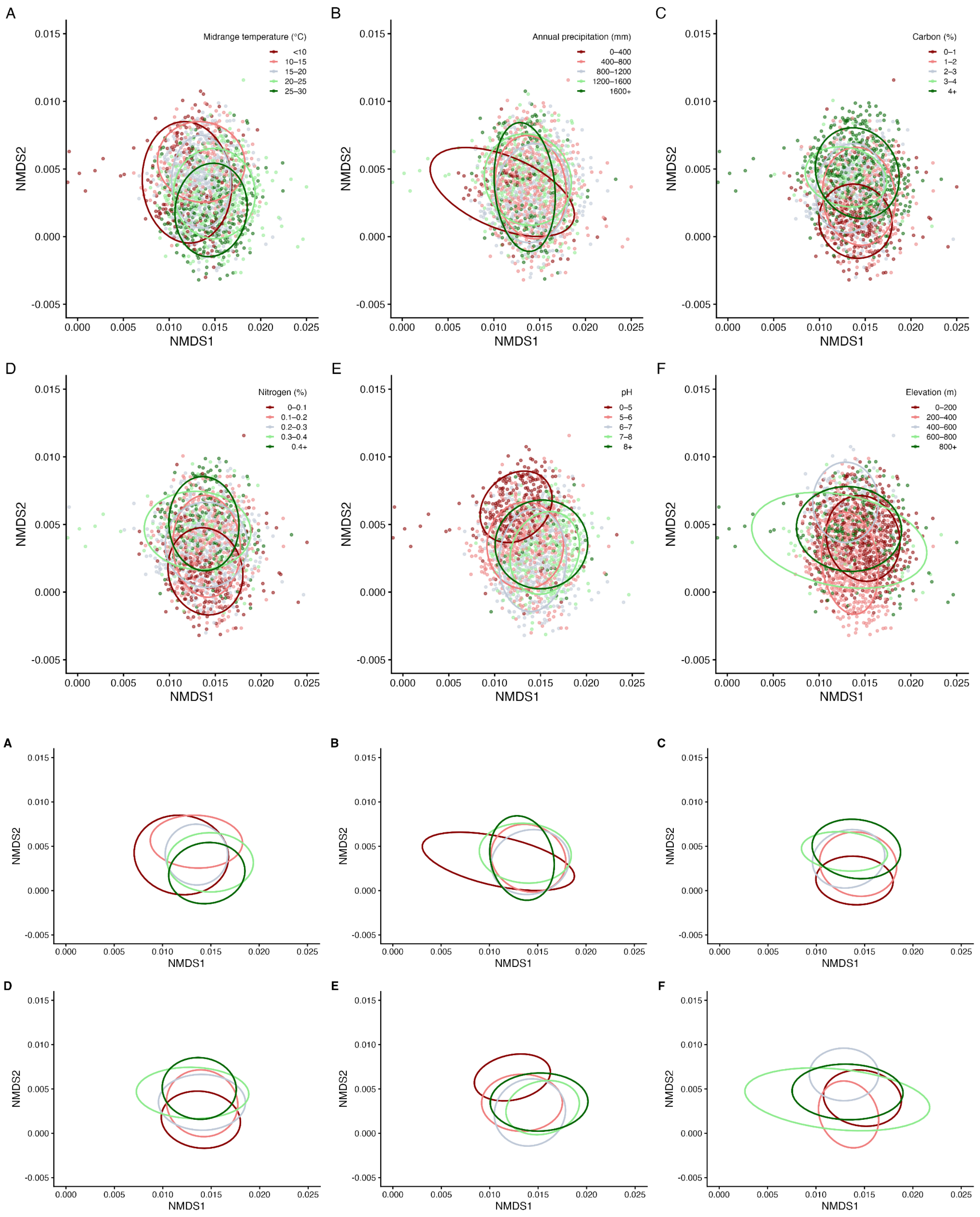

Fig. S8 - Environmental structuring of *Legionella* community composition. NMDS ordination of *Legionella* community composition based on Bray-Curtis dissimilarities calculated from *Legionella* ASV relative abundances. Samples are colored according to environmental bins for midrange temperature, annual precipitation, soil carbon (C), soil nitrogen (N), soil pH and elevation. Ellipses represent 95% confidence intervals around the centroid of samples within each environmental bin.

bioRxiv preprint doi: https://doi.org/10.64898/2026.03.10.710744; this version posted March 11, 2026. The copyright holder for this preprint (which was not certified by peer review) is the author/funder, who has granted bioRxiv a license to display the preprint in perpetuity. It is made available under aCC-BY 4.0 International license.

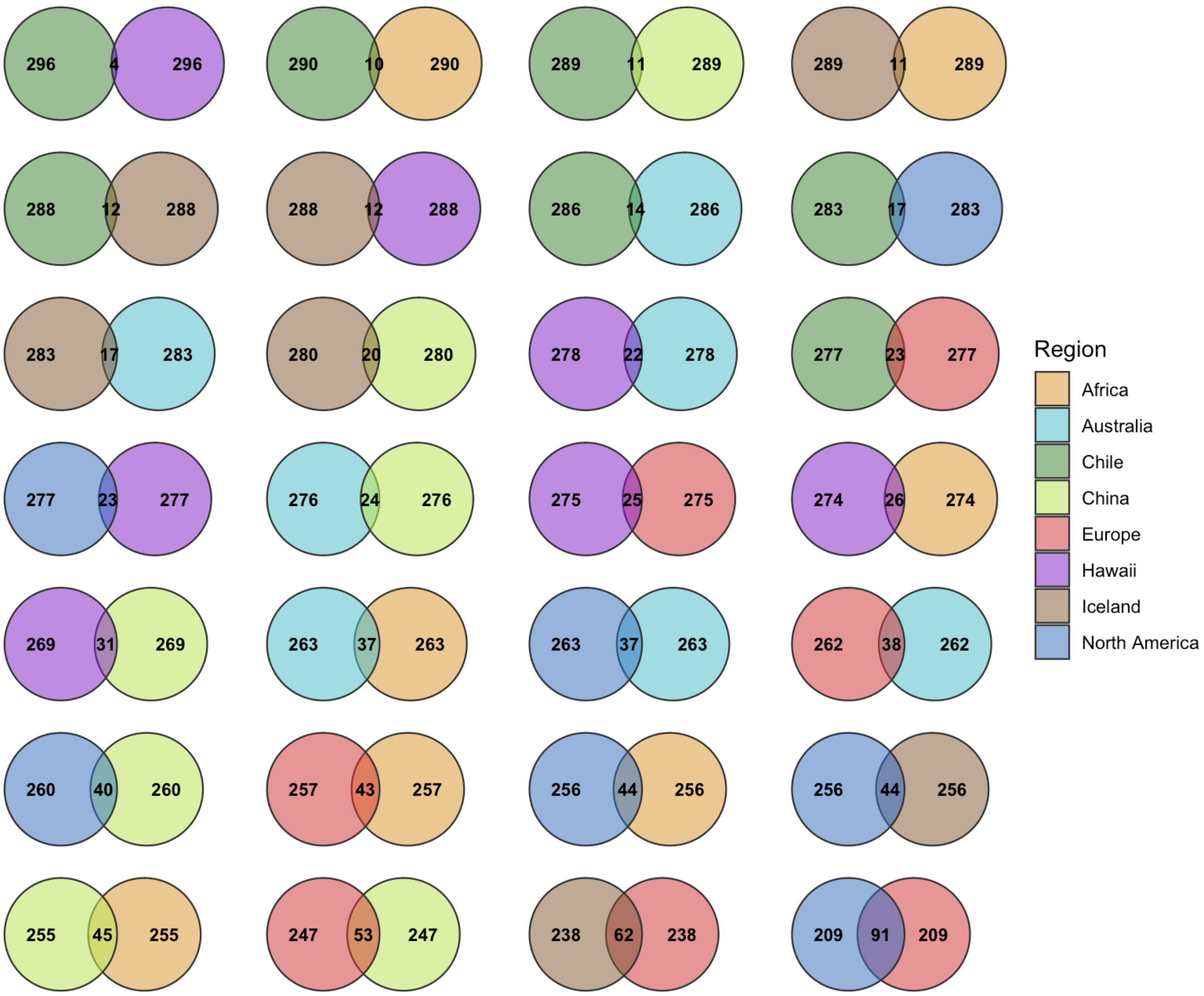

Fig. S9 - Pairwise overlap of dominant *Legionella* ASVs across regions. Venn diagrams illustrate overlap among the 300 most abundant *Legionella* ASVs for each pairwise comparison of the eight regions. Colors indicate regions, and numbers represent the number of dominant ASVs unique to each region (outer sections) or shared between regions (center). Comparisons are arranged from lowest overlap (upper left) to highest overlap (bottom right).

bioRxiv preprint doi: https://doi.org/10.64898/2026.03.10.710744; this version posted March 11, 2026. The copyright holder for this preprint (which was not certified by peer review) is the author/funder, who has granted bioRxiv a license to display the preprint in perpetuity. It is made available under aCC-BY 4.0 International license.

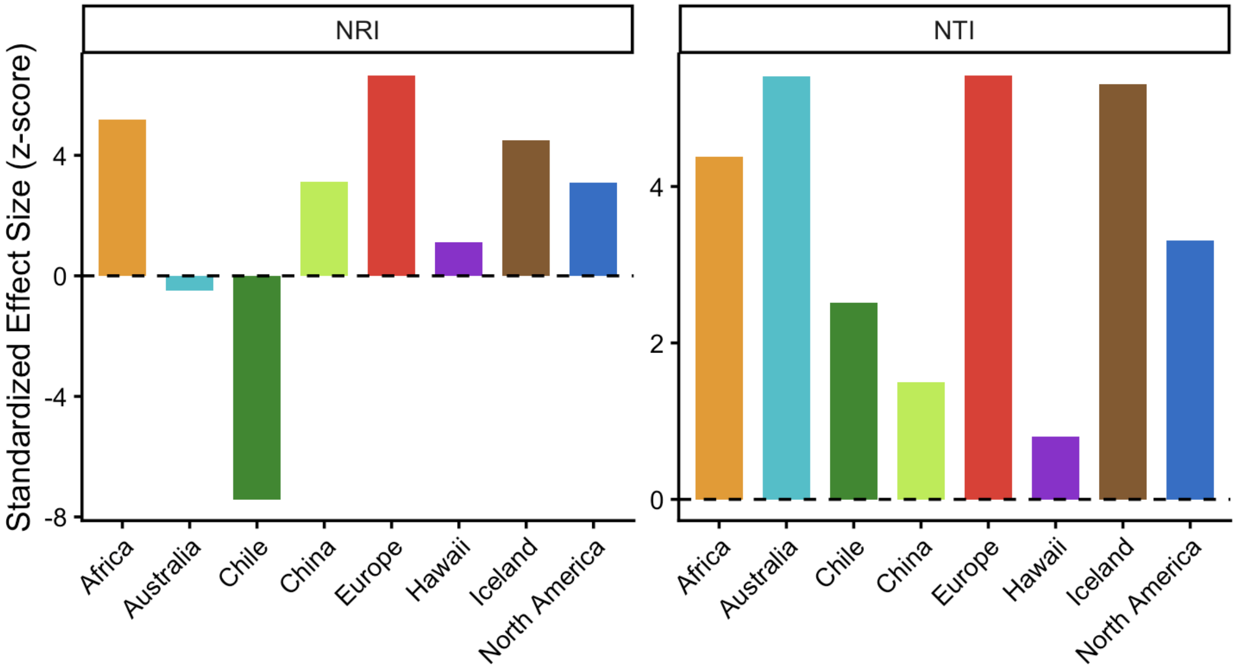

Fig. S10 - Phylogenetic structure of abundant *Legionella* ASVs across global regions. Net Relatedness Index (NRI) and Nearest Taxon Index (NTI) were calculated as standardized effect sizes (SES z-scores) relative to null model communities. Positive values indicate phylogenetic clustering, whereas negative values indicate phylogenetic overdispersion. Bars are colored by geographic region.

bioRxiv preprint doi: https://doi.org/10.64898/2026.03.10.710744; this version posted March 11, 2026. The copyright holder for this preprint (which was not certified by peer review) is the author/funder, who has granted bioRxiv a license to display the preprint in perpetuity. It is made available under aCC-BY 4.0 International license.

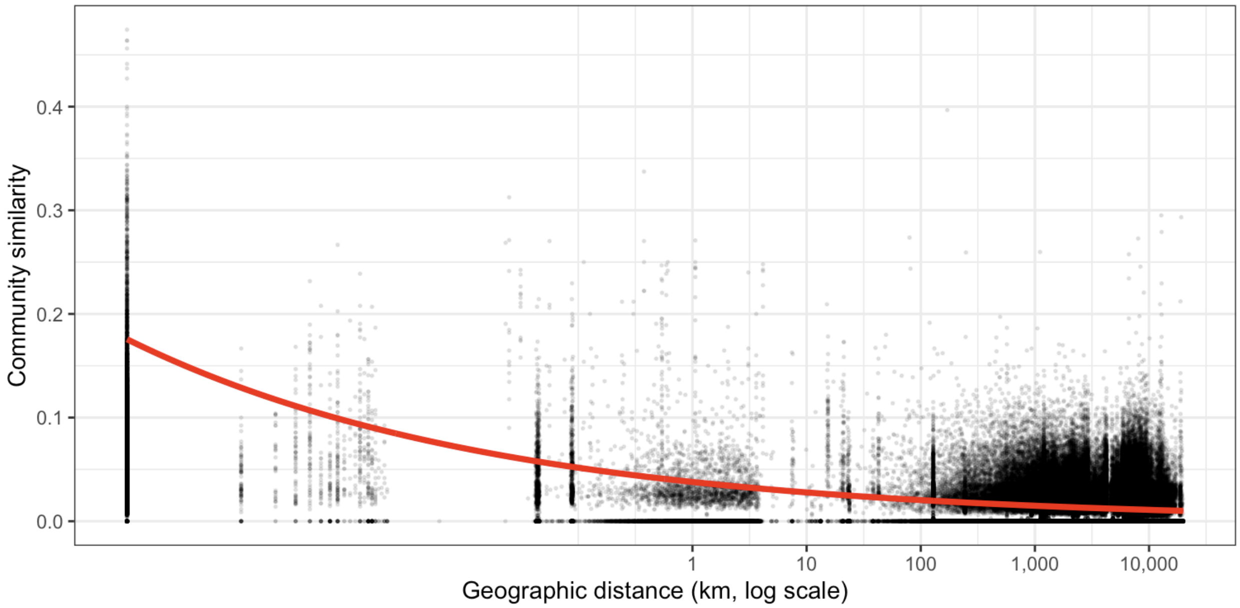

Fig. S11 - Distance-decay relationship of *Legionella* community composition. Pairwise community similarity among samples (1-Bray-Curtis dissimilarity) is plotted against geographic distance (km). Points represent pairwise comparisons between samples, and the red line indicates the fitted distance-decay relationship.

bioRxiv preprint doi: https://doi.org/10.64898/2026.03.10.710744; this version posted March 11, 2026. The copyright holder for this preprint (which was not certified by peer review) is the author/funder, who has granted bioRxiv a license to display the preprint in perpetuity. It is made available under aCC-BY 4.0 International license.

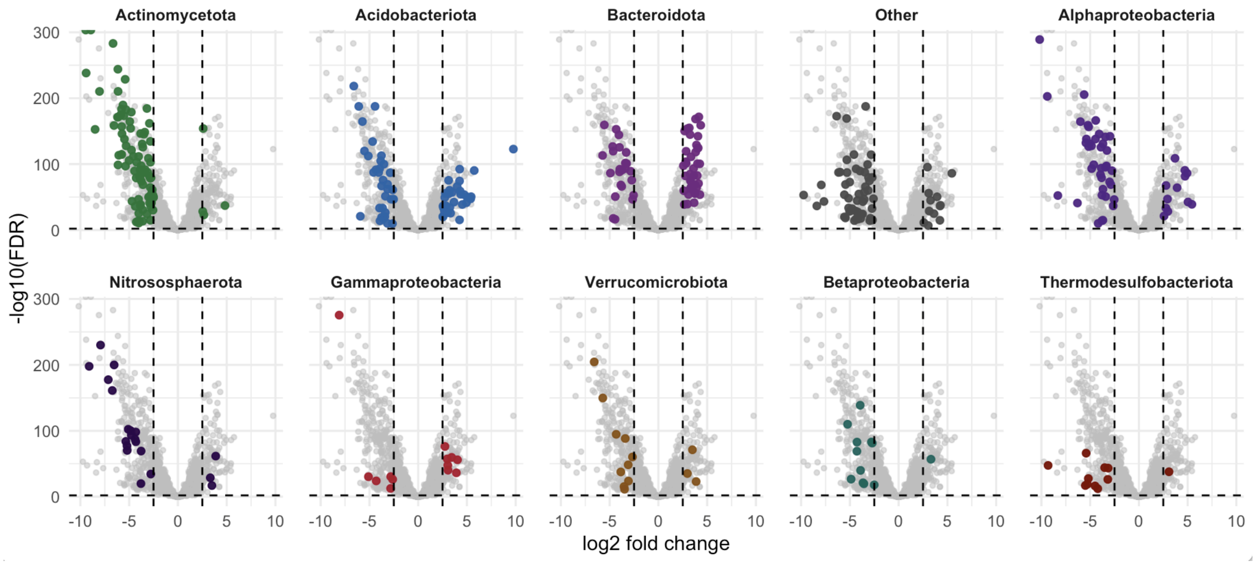

Fig. S12 - Differentially abundant microbial taxa associated with *Legionella* abundance. Volcano plot showing microbial community features (97% OTUs) enriched in *Legionella*-rich versus *Legionella*-poor samples. Samples were classified into upper (>0.062%) and lower (<0.009%) quartiles of *Legionella* relative abundance. Points represent OTUs with $\log_2$ fold change on the x-axis and $-\log_{10}$(FDR) on the y-axis. Positive fold changes indicate enrichment in *Legionella*-rich samples, whereas negative fold changes indicate enrichment in *Legionella*-poor samples. Panels show major taxonomic groups, and colored points indicate OTUs significantly enriched in either direction ($|\log_2$ fold change$| \geq 2.5$, FDR < 0.01).

bioRxiv preprint doi: https://doi.org/10.64898/2026.03.10.710744; this version posted March 11, 2026. The copyright holder for this preprint (which was not certified by peer review) is the author/funder, who has granted bioRxiv a license to display the preprint in perpetuity. It is made available under aCC-BY 4.0 International license.

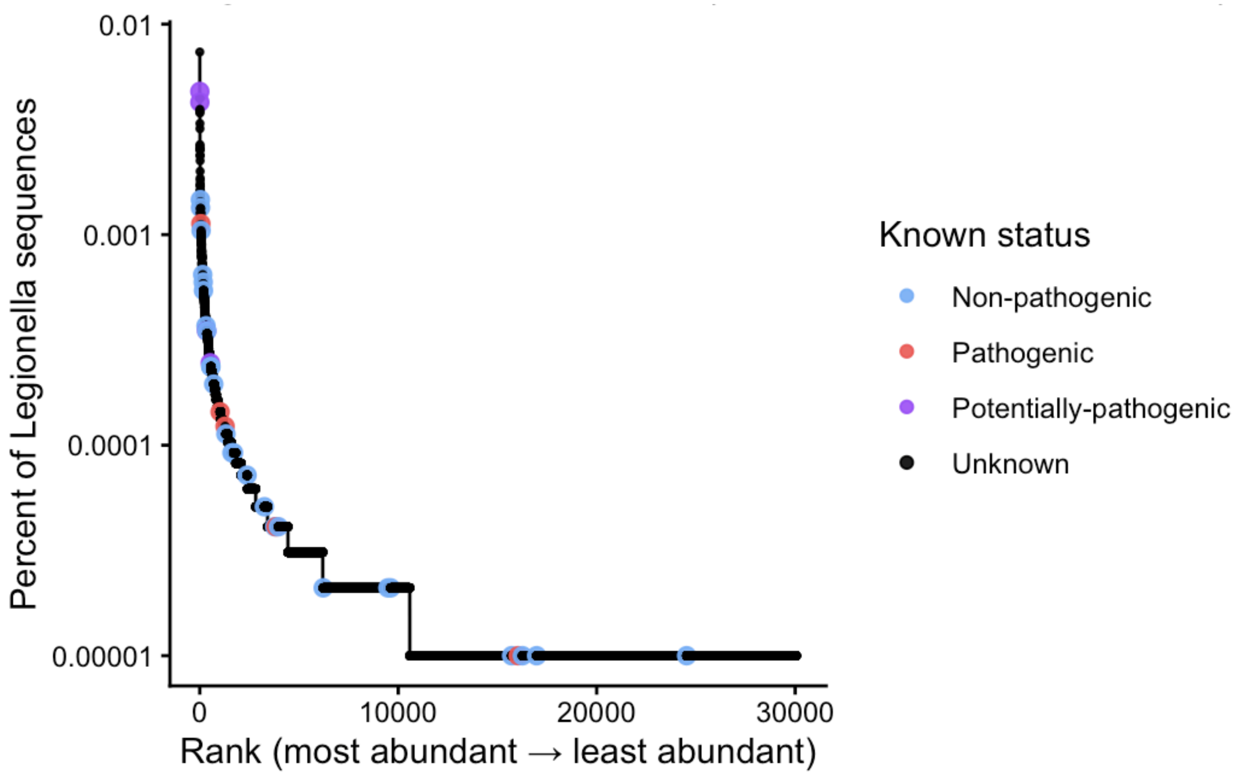

Fig. S13 - Rank abundance distribution of *Legionella* ASVs. Rank abundance plot showing the relative abundance of all *Legionella* ASVs recovered. ASVs are ordered from most abundant to least abundant along the x-axis, and the y-axis indicates the percentage of total *Legionella* sequences represented by each ASV. Points are colored according to pathogenic status based on matches to characterized species (non-pathogenic, pathogenic, potentially pathogenic, or unknown).

bioRxiv preprint doi: https://doi.org/10.64898/2026.03.10.710744; this version posted March 11, 2026. The copyright holder for this preprint (which was not certified by peer review) is the author/funder, who has granted bioRxiv a license to display the preprint in perpetuity. It is made available under aCC-BY 4.0 International license.

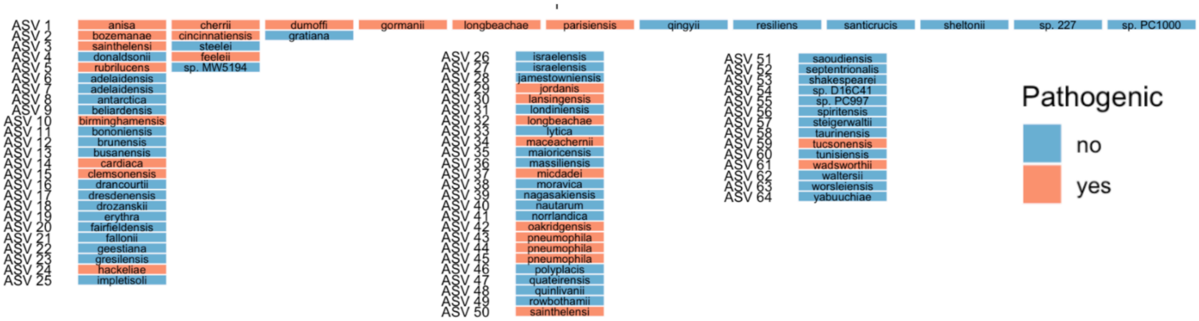

Fig. S14 - Clustering of characterized *Legionella* species based on 16S rRNA gene sequence identity. Clustering of characterized *Legionella* species based on the full 515F-806R 16S rRNA gene amplicon region. Species sharing identical sequences across this region are grouped into the same ASV. Boxes are colored according to whether the species is a known human pathogen.
